# Evaluation of an orthodontic adhesive containing 2 wt% nano-hydroxyapatite synthesized from a biogenic calcium precursor: antibacterial activity, calcium ion concentration, and enamel demineralization

**DOI:** 10.2340/biid.v13.46106

**Published:** 2026-05-20

**Authors:** Gufa Bagus Pamungkas, Eddy Heriyanto Habar, Maria Tanumihardja, Rasmidar Samad, Stang, Eka Erwansyah, Nurlindah Hamrun

**Affiliations:** a Doctoral Program, Faculty of Dentistry, Hasanuddin University, Makassar, Indonesia; b Department of Orthodontics, Faculty of Dentistry, Hasanuddin University, Makassar, Indonesia; c Department of Conservative Dentistry, Faculty of Dentistry, Hasanuddin University, Makassar, Indonesia; d Department of Public Health Dentistry, Faculty of Dentistry, Hasanuddin University, Makassar, Indonesia; e Department of Biostatistics, Faculty of Public Health, Hasanuddin University, Makassar, Indonesia; f Department of Oral Biology, Faculty of Dentistry, Hasanuddin University, Makassar, Indonesia

**Keywords:** Nano-hydroxyapatite, orthodontic adhesive, *Amusium pleuronectes*, antibacterial activity, enamel demineralization, biogenic precursor

## Abstract

**Objective:**

Enamel demineralization and white spot lesions remain common adverse effects during fixed orthodontic treatment, primarily due to plaque accumulation around brackets and the limited functionality of conventional orthodontic adhesives. This study aimed to evaluate the effect of incorporating 2 wt% nano-hydroxyapatite (nHAp), synthesized using a biogenic calcium precursor derived from Asian moon scallop (*Amusium pleuronectes*) shells, on antibacterial activity, calcium ion concentration, and enamel demineralization.

**Materials and Methods:**

nHAp powder was synthesized from scallop shell-derived calcium carbonate via calcination and precipitation methods and incorporated into a commercial orthodontic adhesive under controlled conditions. Antibacterial activity against *Streptococcus mutans* was assessed using a disc diffusion assay. Calcium ion concentration in the immersion medium was measured using UV-Vis spectrophotometry (λ = 515 nm) with a murexide indicator. Enamel demineralization adjacent to bracket interfaces was evaluated using a standardized scoring system. Statistical analysis was performed using appropriate parametric and non-parametric tests with significance set at *p* < 0.05.

**Results:**

The nHAp-modified adhesive showed significantly greater antibacterial activity compared with the control group (*p* < 0.05). The experimental group demonstrated higher calcium ion concentration in the immersion medium than the control group. Enamel demineralization scores were significantly lower in the experimental group.

**Conclusion:**

Within the limitations of this in vitro study, incorporation of 2 wt% nano-hydroxyapatite synthesized using a biogenic calcium precursor was associated with increased antibacterial activity, higher calcium ion concentration in the surrounding medium, and reduced enamel demineralization. Further studies are required to evaluate mechanical properties and clinical applicability.

## Introduction

Fixed orthodontic treatment remains a widely used approach for correcting malocclusion and dentofacial discrepancies. However, it is frequently associated with enamel demineralization at the bracket-adhesive-enamel interface, primarily due to plaque accumulation and acidogenic bacterial activity around orthodontic appliances [1]. The presence of cariogenic microorganisms, particularly *Streptococcus mutans*, contributes to localized pH reduction, leading to mineral loss and the formation of white spot lesions (WSLs), even in patients with otherwise satisfactory oral hygiene [2]. Conventional orthodontic adhesives are primarily designed to provide adequate mechanical retention and do not possess intrinsic antibacterial or remineralizing properties. As a result, they offer limited protection against demineralization under cariogenic conditions [3]. This limitation has prompted the development of modified adhesive systems incorporating functional fillers to improve their biological performance.

Nano-hydroxyapatite (nHAp) has emerged as a promising additive in dental materials due to the chemical similarity to the mineral phase of enamel and its high surface area, which enhances reactivity [4]. The incorporation of nHAp into resin-based materials has been reported to be associated with ion exchange processes, mineral deposition, and improved interfacial interactions with enamel [5, 6]. Although hydroxyapatite is not inherently a strong antibacterial agent, several mechanisms have been proposed to explain the potential indirect antibacterial effects, including inhibition of bacterial adhesion, modification of surface charge interactions, and alteration of local ionic environments that may influence biofilm formation [7].

In recent years, increasing attention has been directed toward the use of sustainable and biogenic sources as calcium precursors for hydroxyapatite synthesis. Marine shells, including those of mollusks, are primarily composed of calcium carbonate and can be converted into hydroxyapatite through calcination and chemical precipitation processes [8]. Following these processes, the resulting nHAp is chemically comparable to synthetic hydroxyapatite; therefore, its functional properties should be attributed to its physicochemical characteristics rather than its biological origin [9].

The Asian moon scallop (*Amusium pleuronectes*) shell represents a readily available marine resource with high calcium content, making it a potential alternative precursor for hydroxyapatite synthesis [10]. The selection in this study is based on availability, sustainability, and suitability as a calcium source for controlled hydroxyapatite production [11]. However, the application in orthodontic adhesive systems remains limited in the current literature.

Despite growing interest in nHAp-modified orthodontic materials, studies that comprehensively evaluate antibacterial activity, calcium ion concentration in the surrounding medium, and enamel demineralization remain limited. Furthermore, clearer differentiation between material origin and demonstrated functional performance is needed.

Therefore, the present study aimed to evaluate the properties of an orthodontic adhesive incorporated with 2 wt% nHAp synthesized using a biogenic calcium precursor derived from *Amusium pleuronectes* shells. The 2 wt% concentration was selected based on previous evidence indicating that this proportion maintains optimal viscosity and handling characteristics [12, 13]. The modified adhesive was compared with an unmodified control in terms of antibacterial activity against *Streptococcus mutans*, calcium ion concentration in the immersion medium, and enamel demineralization.

## Materials and methods

This laboratory-based experimental study was conducted following approval from the Research Ethics Committee of the Faculty of Dentistry – Dental Hospital, Hasanuddin University (Letter No. 007/KEPK FKG-RSGMP UH/EA/I/2025). The study consisted of two groups: (1) unmodified orthodontic adhesive (control) and (2) adhesive modified with 2 wt% nHAp.

Sample size determination was based on ensuring adequate replication for each independent experiment, consistent with previous in vitro dental materials studies [10, 14]. Five specimens per group were used for antibacterial testing, while 16 specimens per group were allocated for calcium ion concentration analysis and enamel demineralization evaluation. Each experimental outcome was conducted and analyzed independently.

### Synthesis of nano-hydroxyapatite

nHAp was synthesized using a wet precipitation method adapted from previously reported protocols [15]. Shells of *Amusium pleuronectes* were cleaned, boiled to remove organic matter, dried, and ground into fine powder. The powder was calcined at 900°C for 3 h to convert calcium carbonate (CaCO₃) into calcium oxide (CaO). The CaO was then hydrated to form calcium hydroxide (Ca(OH)₂). A 0.3 M phosphoric acid (H₃PO₄) solution was added dropwise into a 0.5 M Ca(OH)₂ suspension under continuous magnetic stirring at 80°C, maintaining a Ca/P molar ratio of 1.67. The mixture was aged for 24 h to allow complete precipitation. The precipitate was filtered, washed with deionized water, and dried at 100°C. A final calcination step at 800°C for 2 h was performed to obtain crystalline nHAp powder.

**Figure 1:**
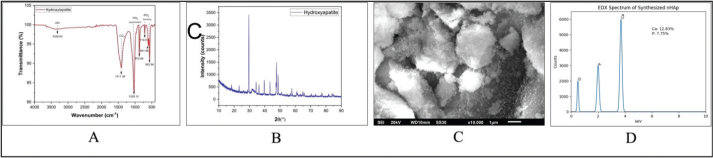
Characterization of synthesized nano-hydroxyapatite (nHAp). (A) FTIR spectrum showing characteristic phosphate (PO₄^3-^) vibrational bands and hydroxyl (OH^3–^) groups, confirming hydroxyapatite formation. (B) XRD pattern exhibiting diffraction peaks consistent with the crystalline hydroxyapatite phase. (C) SEM micrograph at 10,000× magnification revealing relatively uniform particle distribution with nanoscale morphology. (D) EDX spectrum confirming elemental composition (Ca, P, and O), with Ca/P ratio consistent with hydroxyapatite.

The synthesized nHAp was characterized using Fourier Transform Infrared Spectroscopy (FTIR) (PerkinElmer Spectrum 2, USA), X-ray Diffraction (XRD) (Shimadzu XRD-7000, Japan; Cu-Kα radiation, λ = 1.5406 Å, 2θ range: 20–60°), and Scanning Electron Microscopy with Energy Dispersive X-ray Spectroscopy (SEM–EDX) (JEOL JSM-6510LA, Japan; accelerating voltage: 20 kV). Representative spectra and micrographs are presented in Figure 1.

### Preparation of experimental adhesive

The experimental adhesive was prepared by incorporating 2 wt% nHAp into a commercial orthodontic adhesive (Transbond™ XT, 3M Unitek, USA). The required mass of nHAp was calculated relative to the total mass of adhesive used. A predetermined amount of adhesive was dispensed onto a glass slab and weighed using an analytical balance (accuracy ±0.0001 g). The corresponding mass of nHAp (2 wt%) was then added.

Mixing was performed by manual spatulation using a glass spatula for approximately 10 min under light-protected conditions. The material was repeatedly folded and spread to promote particle distribution. It is acknowledged that manual mixing may result in non-uniform nanoparticle dispersion and potential agglomeration. The absence of high-energy mixing techniques (e.g. planetary or ultrasonic mixing) represents a limitation and may influence material homogeneity. To minimize variability, all samples were prepared under standardized conditions (same operator, mixing time, and ambient temperature).

### Antibacterial activity test

Antibacterial activity was evaluated against *Streptococcus mutans* (ATCC 25175) using a disc diffusion method [16]. The bacterial suspension was adjusted to a 0.5 McFarland standard (~1 × 10⁸ CFU/mL). Mueller-Hinton agar supplemented with 5% sheep blood was inoculated uniformly. Disc-shaped specimens (6 mm diameter × 2 mm thickness) were prepared and sterilized using ultraviolet irradiation for 30 min per side, following previously described protocols [17]. Five specimens per group were placed onto the inoculated agar plates and incubated at 37°C for 24 h under microaerophilic conditions. The diameter of the inhibition zone was measured using a digital caliper. Measurements were performed by two independent examiners, and inter-examiner reliability was assessed using Cohen’s kappa coefficient.

### Calcium ion concentration measurement

Calcium ion concentration in the immersion medium was evaluated to assess the presence of calcium ions in the surrounding solution. Specimens (*n* = 16 per group) were immersed individually in 10 mL phosphate-buffered saline (PBS, pH 7.4) and incubated at 37°C. After 24 h, the immersion solution was collected for analysis. Calcium ion concentration was determined using a UV-Vis spectrophotometer (Shimadzu UV-1240, Japan) at a wavelength of 515 nm with a murexide indicator. Calibration was performed using standard CaCl₂ solutions, and results were expressed in ppm [18]. The 24-h time point was selected to evaluate initial calcium ion concentration in the medium. It is acknowledged that multiple time-point measurements (e.g. 7, 14, and 21 days) would provide a more comprehensive evaluation of ion dynamics and should be considered in future studies.

### Enamel demineralization analysis

Thirty-two extracted human premolars were collected and randomly assigned into two groups (*n* = 16 per group). Extracted teeth used in this study were collected from dental clinics as discarded biological material. The samples were fully anonymized and contained no patient identifiers. In accordance with institutional guidelines, the use of such materials does not require informed consent or ethical approval. Teeth were cleaned and stored in 0.1% thymol solution until use. Orthodontic brackets were bonded using either control or experimental adhesive according to the manufacturer’s instructions. The specimens were immersed in a demineralizing solution (pH 4.5) and incubated at 37°C for 21 days, following established in vitro protocols for simulating enamel demineralization and WSL formation [19].

**Figure 2:**
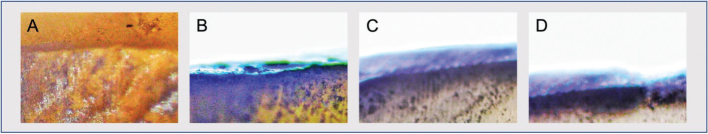
Enamel demineralization scoring system (A. Score 0: No visible demineralization; B. Score 1: Demineralization limited to the prismless outer enamel layer; C. Score 2: Demineralization extending into the inner enamel without surface disrupt; D. Score 3: Severe demineralization extending through the inner enamel with surface damage).

Following incubation, specimens were examined using a stereomicroscope (Olympus SZ61, Japan) at 40× magnification. Enamel demineralization was assessed using a standardized four-point scoring system (Figure 2), as described in previous studies [20]. Two calibrated examiners performed blinded evaluations. Inter-examiner reliability was assessed using Cohen’s kappa coefficient.

### Statistical analysis

Statistical analyses were performed using IBM SPSS Statistics version 29.0 (IBM Corp., Armonk, NY, USA), with the level of significance set at *p* < 0.05. Data normality was assessed using the Shapiro-Wilk test. For normally distributed data, comparisons between groups were performed using an independent t-test. For data that were not normally distributed, the Mann-Whitney U test was applied.

Antibacterial activity and calcium ion concentration data were analyzed according to the results of the normality test. Enamel demineralization scores, being ordinal in nature, were analyzed using the Mann-Whitney U test.

## Result

**Figure 3:**
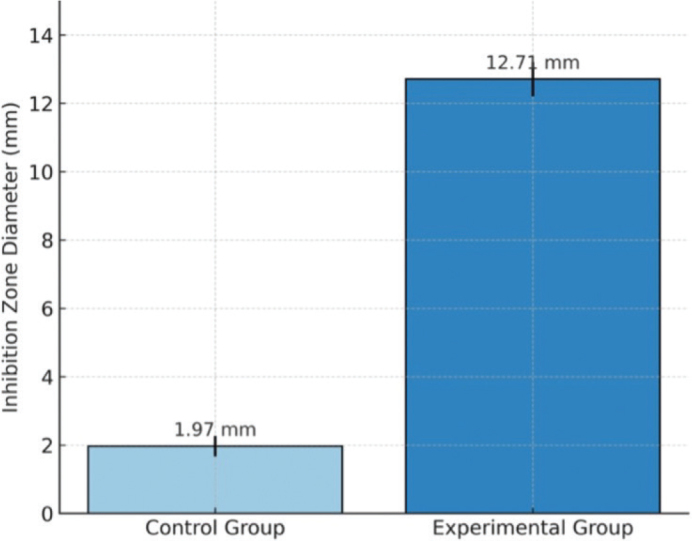
Mean inhibition zone diameter (mm) against *Streptococcus mutans*.

The antibacterial test results (Figure 3) showed that the experimental group exhibited a larger inhibition zone compared to the control group. The mean inhibition zone diameter in the experimental group was 12.71 ± 2.07 mm, while the control group showed 1.97 ± 2.04 mm. Normality testing using the Shapiro-Wilk test for the experimental and control group indicated that the data were normally distributed with *p* = 0.200 and *p* = 0.672, respectively. Accordingly, an independent t-test revealed a statistically significant difference between the groups (*p* = 0.000).

**Figure 4:**
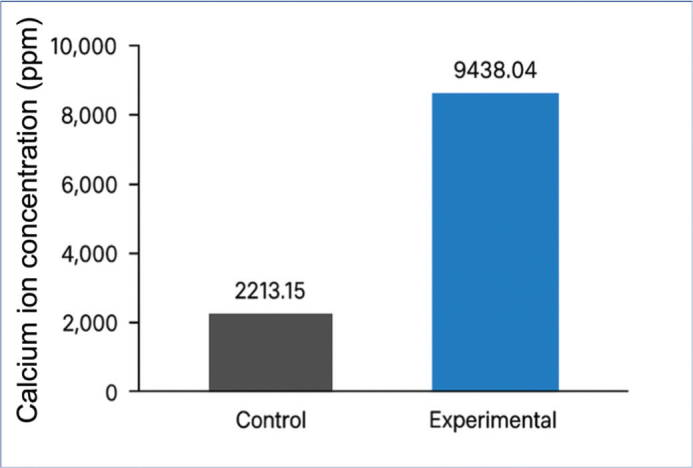
Mean calcium ion concentration (ppm) in the immersion medium for control and nHAp-modified adhesive groups.

Calcium ion concentration in the immersion medium, measured using a UV-Vis spectrophotometer at 515 nm with a murexide indicator (Figure 4), was 9438.04 ± 184.18 ppm in the experimental group and 2213.15 ± 69.77 ppm in the control group. Normality testing using the Shapiro-Wilk test for the experimental and control group indicated that the data were normally distributed with *p* = 0.349 and *p* = 0.111, respectively. Statistical analysis used an independent t-test showed that the difference between the groups was significant (*p* = 0.000).

The evaluation of enamel demineralization around orthodontic brackets (Table 1) showed that 75% of samples in the experimental group had a score of 0 (no demineralization). In contrast, the control group showed the highest proportion of score 1 (37.5%). The distribution of scores between groups was analyzed using the Mann-Whitney test and showed a statistically significant difference (*p* = 0.011).

**Table 1:** The results of enamel demineralization observation around the orthodontic brackets.

Score	Experimental (sample)	Percentage	Control (sample)	Percentage
0	12	75.00	5	31.25
1	3	18.75	6	37.50
2	1	6.25	4	25.00
3	0	0.00	1	6.25

## Discussion

The present in vitro investigation demonstrated that incorporation of 2 wt% nHAp, synthesized using *Amusium pleuronectes* shell as a calcium precursor, into a commercial orthodontic adhesive was associated with increased antibacterial activity against *Streptococcus mutans*, higher calcium ion concentration in the immersion medium, and reduced enamel demineralization adjacent to bonded brackets. These findings suggest that the addition of low concentrations of nHAp may impart functional modifications to orthodontic adhesives [13].

The enhanced antibacterial effect observed in the modified adhesive may be attributed to physicochemical interactions between nHAp particles and bacterial cells [21]. Although hydroxyapatite is not inherently antibacterial, previous studies have suggested that nanoscale particles may influence bacterial adhesion and biofilm formation through surface charge interactions and increased surface area [22]. In addition, the presence of calcium ions in the surrounding medium may alter local environmental conditions and affect bacterial metabolic activity [23]. Therefore, the observed antibacterial effect is likely multifactorial and may involve both surface-related interactions and ionic effects rather than a direct bactericidal mechanism.

The calcium ion measurements showed a higher concentration in the experimental group compared to the control group. This finding suggests that the presence of nHAp may be associated with increased calcium concentration in the surrounding medium [24]. However, it should be emphasized that the measured calcium values represent the total calcium detected in the immersion solution and do not necessarily reflect intrinsic calcium release solely from the adhesive matrix [25]. Potential contributions from the immersion medium, measurement method, or experimental conditions should therefore be considered when interpreting these results. Furthermore, the calcium concentrations observed in the present study are relatively higher than those reported in previous studies on calcium-phosphate-based dental materials, which typically demonstrate lower ion concentrations. This discrepancy may be attributed to differences in analytical techniques (e.g. Ultraviolet-Visible (UV-Vis) spectrophotometry vs. Inductively Coupled Plasma (ICP)-based methods), nanoparticle surface area and solubility, or variations in experimental design, including immersion conditions and sample preparation. Therefore, direct comparison with previous studies should be interpreted with caution [26].

The stereomicroscopic evaluation demonstrated that 75% of samples in the experimental group exhibited no visible demineralization, whereas the control group showed a higher proportion of superficial lesions. This observation suggests that the presence of nHAp may influence mineral dynamics at the enamel–adhesive interface [20]. The combined effects of increased calcium ion availability and reduced bacterial activity may contribute to limiting enamel mineral loss under acidic conditions. Similar trends have been reported in studies evaluating calcium-phosphate-containing dental materials, although variations in experimental design make direct comparison difficult [23, 27].

The important to clarify that the nHAp used in this study, although derived from *Amusium pleuronectes* shells, undergoes calcination and chemical processing to form hydroxyapatite. As a result, the final material is chemically comparable to synthetic hydroxyapatite, and its functional properties should be attributed to its physicochemical characteristics rather than its biological origin. Therefore, any observed effects should be interpreted based on material behavior rather than assumed inherent bioactivity from the source material [28–30].

The selection of 2 wt% nHAp was based on previous reports indicating that low nanoparticle loading levels can provide functional benefits while minimizing adverse effects on material properties. Higher filler concentrations may increase the risk of nanoparticle agglomeration and negatively affect resin viscosity, degree of conversion, and mechanical performance [10, 12]. However, mechanical properties such as shear bond strength were not evaluated in the present study and should be included in future investigations to confirm clinical applicability.

Several limitations should be acknowledged. The antibacterial assessment was based on a disc diffusion method, which primarily reflects inhibition of planktonic bacterial growth and does not fully represent complex oral biofilm conditions [31]. Additionally, calcium ion measurement was performed at a single time point, limiting the ability to assess release kinetics over time. Future studies should incorporate multiple time intervals and more sensitive analytical techniques, such as inductively coupled plasma methods, to provide a more comprehensive evaluation. Furthermore, the manual mixing technique used for incorporating nHAp may result in variability in particle dispersion, which could influence material performance. Future research should focus on evaluating nanoparticle dispersion using standardized mixing methods, assessing mechanical properties, including shear bond strength, and employing more clinically relevant models such as multispecies biofilms and pH-cycling systems. In addition, in situ or clinical studies are necessary to determine whether the observed in vitro effects translate into meaningful reductions in WSL formation during orthodontic treatment.

## Conclusion

Within the limitations of this in vitro study, the incorporation of 2 wt% nHAp, synthesized using *Amusium pleuronectes* shell as a calcium precursor, into an orthodontic adhesive was associated with increased antibacterial activity against *Streptococcus mutans*, higher calcium ion concentration in the immersion medium, and reduced enamel demineralization. These findings suggest that the addition of low concentrations of nHAp may provide functional modifications to orthodontic adhesives. However, further studies, including mechanical property evaluation and clinically relevant models, are required to confirm its applicability in orthodontic practice.

## Data Availability

The data supporting the findings of this study are available from the corresponding author upon reasonable request. Due to confidentiality agreements and institutional policies, the raw data sets are not publicly accessible but can be provided to qualified researchers for verification and further research purposes.

## References

[1] Shaik JA, Reddy RK. Review article prevention and treatment of white spot lesions in orthodontic patients. Contemp Clin Dent. 2017;8(September):11–19. 10.4103/ccd.ccd_216_17 28566845 PMC5426141

[2] Azaripour A, Willershausen I, Hassan M, Ebenezer S, Willershausen B. Oral hygiene and dietary habits in adolescents with fixed orthodontic appliances: a cross-sectional study. J Contemp Dent Pract. 2016;17(3):179–83. 10.5005/jp-journals-10024-1824 27207195

[3] Hamdi K, Elsebaai A, Abdelshafi MA, Hamama HH. Remineralization and anti-demineralization effect of orthodontic adhesives on enamel surrounding orthodontic brackets: a systematic review of in vitro studies. BMC Oral Health. 2024;24(1):1446. 10.1186/s12903-024-05237-y 39609782 PMC11603835

[4] Florea AD, Pop LC, Benea HRC, et al. Remineralization induced by biomimetic hydroxyapatite toothpastes on human enamel. Biomimetics. 2023;8(6):1–19. 10.3390/biomimetics8060450 PMC10604461 37887581

[5] Memarpour M, Shafiei F, Rafiee A, Soltani M, Dashti MH. Effect of hydroxyapatite nanoparticles on enamel remineralization and estimation of fissure sealant bond strength to remineralized tooth surfaces: an in vitro study. BMC Oral Health. 2019;19(1):1–14. 10.1186/s12903-019-0785-6 31138191 PMC6540542

[6] Valluri BP, Kanumuri MV, Sajjan G, Rajulapati KS, Penmatsa VKV, Mavidi JB. Effect of nano-hydroxyapatite incorporation on the immediate and long-term bond stability of a one-step self-etch adhesive. J Dent Mater Tech. 2025;14(2):57–63. 10.22038/jdmt.2025.85728.1781

[7] Ibrahim AZ, Hussein AS, Said Gulam Khan HB, Ghazali N. Antibacterial activity of microwave synthesized hydroxyapatite against cariogenic bacteria: a preliminary study. Saudi Dent J. 2024;36(8):1117–22. 10.1016/j.sdentj.2024.06.004 39176152 PMC11337952

[8] Muntean FL, Olariu I, Marian D, et al. Hydroxyapatite from mollusk shells: characteristics, production, and potential applications in dentistry. Dent J (Basel). 2024;12(12):1–24. 10.3390/dj12120409 PMC11674191 39727466

[9] Syafaat FY, Yusuf Y. Influence of ca/p concentration on hydroxyapatite (Hap) from Asian moon scallop shell (Amusium pleuronectes). Int J Nanoelectron Mater. 2019;12(3):357–62.

[10] Pamungkas GB, Habar EH, Tanumihardja M. In vitro evaluation of enamel bond strength of an orthodontic adhesive enhanced with 2 wt% nano-hydroxyapatite derived from Asian moon scallop (Amusium pleuronectes). Biomater Investig Dent. 2025;12:233–8. 10.2340/biid.v12.45130 PMC12742223 41458883

[11] Soffa FB, Pratama IS, Dharmawati V, et al. Asian moon scallop (Amusium pleuronectes) for indonesia: an overview from a wild population and farming system. Fish Aquatic Sci. 2024;27(11):709–27. 10.47853/FAS.2024.e67

[12] Hasan LA. Evaluation the properties of orthodontic adhesive incorporated with nano-hydroxyapatite particles. Saudi Dent J. 2021;33(8):1190–6. 10.1016/j.sdentj.2021.01.001 34938065 PMC8665179

[13] Noworyta M, Topa-Skwarczyńska M, Jamróz P, et al. Influence of the type of nanofillers on the properties of composites used in dentistry and 3d printing. Int J Mol Sci. 2023;24(13):1-23. 10.3390/ijms241310549 PMC10341704 37445729

[14] Mahardhika MH, Karunia D, Pudyani PS, Alhasyimi AA. Effect of a desensitizing agent on shear bond strength of ceramic bracket on previously bleached teeth. Appl Sci (Switzerland). 2023;13(14):1-11. 10.3390/app13148351

[15] Cahyaningrum SE, Herdyastuty N, Devina B, Supangat D. Synthesis and characterization of hydroxyapatite powder by wet pecipitation method. IOP Conf Ser Mater Sci Eng. 2018;299(1):1-6. 10.1088/1757-899X/299/1/012039

[16] Wassel MO, Khattab MA. Antibacterial activity against streptococcus mutans and inhibition of bacterial induced enamel demineralization of propolis, miswak, and chitosan nanoparticles based dental varnishes. J Adv Res. 2017;8(4):387–92. 10.1016/j.jare.2017.05.006 28560054 PMC5443966

[17] Balouiri M, Sadiki M, Ibnsouda SK. Methods for in vitro evaluating antimicrobial activity: a review. J Pharm Anal. 2016;6(2):71–9. 10.1016/j.jpha.2015.11.005 29403965 PMC5762448

[18] Moser JH. The colorimetric determination of calcium with ammonium purpurate. Analytical Chemistry. 1952;24(3):556–559.

[19] ten Cate JM, Duijsters PPE. Alternating demineralization and remineralization of artificial enamel lesions. Caries Res. 1982;16(3):201–10. 10.1159/000260599 6953998

[20] Hennig CL, Löhnert S, Nitzsche A, et al. Influence of different bracket adhesive systems on enamel demineralization – an in vitro study. J Clin Med. 2023;12(13):1–10. 10.3390/jcm12134494 PMC10342608 37445529

[21] Uskoković V, Tang S, Nikolić MG, Marković S, Wu VM. Calcium phosphate nanoparticles as intrinsic inorganic antimicrobials: in search of the key particle property. Biointerphases. 2019;14(3):1-20. 10.1116/1.5090396 PMC6527436 31109162

[22] Pushpalatha C, Gayathri VS, Sowmya SV, et al. Nanohydroxyapatite in dentistry: a comprehensive review. Saudi Dent J. 2023;35(6):741–52. 10.1016/j.sdentj.2023.05.018 37817794 PMC10562112

[23] Pamungkas GB, Karunia D, Suparwitri S. Desensitizing agents’ post-bleaching effect on orthodontic bracket bond strength. Dent J. 2024;45(158):45–9. 10.20473/j.djmkg.v57.i1.p45

[24] Wiryani M, Sujatmiko B, Bikarindrasari R. Effect of duration of application of remineralizing agent casein phosphopeptide-amorphous calcium phosphate fluoride (CPP-ACPF) on enamel hardness. Maj Ked Gi Ind. 2016;2(3):141–146. 10.22146/majkedgiind.11250

[25] Huang SB, Gao SS, Yu HY. Effect of nano-hydroxyapatite concentration on remineralization of initial enamel lesion in vitro. Biomed Mater. 2009;4(3):34104. 10.1088/1748-6041/4/3/034104 19498220

[26] Bin-Jardan LI, Almadani DI, Almutairi LS, et al. Inorganic compounds as remineralizing fillers in dental restorative materials: narrative review. Int J Mol Sci. 2023;24(9):1-26. 10.3390/ijms24098295 PMC10179470 37176004

[27] Nizami MZI, Jindarojanakul A, Ma Q, Lee SJ, Sun J. Advances in bioactive dental adhesives for caries prevention: a state-of-the-art review. J Funct Biomater. 2025;16(11):418. 2025. 10.3390/jfb16110418 41295073 PMC12653816

[28] Chen L, Al-Bayatee S, Khurshid Z, Shavandi A, Brunton P, Ratnayake J. Hydroxyapatite in oral care products – a review. Materials. 2021;14(17):1-20. 10.3390/ma14174865 PMC8432723 34500955

[29] Mohd Pu’ad NAS, Koshy P, Abdullah HZ, Idris MI, Lee TC. Syntheses of hydroxyapatite from natural sources. Heliyon. 2019;5(5):e01588. 10.1016/j.heliyon.2019.e01588 31080905 PMC6507053

[30] Fiume E, Magnaterra G, Rahdar A, Verné E, Baino F. Hydroxyapatite for biomedical applications: a short overview. Ceramics. 2021;4(4):542–63. 10.3390/ceramics4040039

[31] Jain A, Armstrong SR, Banas JA, Qian F, Maia RR, Teixeira EC. Dental adhesive microtensile bond strength following a biofilm-based in vitro aging model. J Appl Oral Sci. 2020;28:1–9. 10.1590/1678-7757-2019-0737 PMC7340208 32609185

